# Different doses of intermittent theta burst stimulation for upper limb motor dysfunction after stroke: a study protocol for a randomized controlled trial

**DOI:** 10.3389/fnins.2023.1259872

**Published:** 2023-10-05

**Authors:** Zhiqing Tang, Tianhao Liu, Ying Liu, Kaiyue Han, Wenlong Su, Jingdu Zhao, Qianqian Chi, Xiaonian Zhang, Hao Zhang

**Affiliations:** ^1^School of Rehabilitation, Capital Medical University, Beijing, China; ^2^China Rehabilitation Research Center, Beijing Bo'ai Hospital, Beijing, China; ^3^University of Health and Rehabilitation Sciences, Qingdao, China; ^4^Cheeloo College of Medicine, Shandong University, Jinan, China

**Keywords:** study protocol, stroke, intermittent theta burst stimulation, upper limb, randomized controlled trial

## Abstract

**Background:**

Upper limb motor recovery is one of the important goals of stroke rehabilitation. Intermittent theta burst stimulation (iTBS), a new type of repetitive transcranial magnetic stimulation (rTMS), is considered a potential therapy. However, there is still no consensus on the efficacy of iTBS for upper limb motor dysfunction after stroke. Stimulus dose may be an important factor affecting the efficacy of iTBS. Therefore, we aim to investigate and compare the effects and neural mechanisms of three doses of iTBS on upper limb motor recovery in stroke patients, and our hypothesis is that the higher the dose of iTBS, the greater the improvement in upper limb motor function.

**Methods:**

This prospective, randomized, controlled trial will recruit 56 stroke patients with upper limb motor dysfunction. All participants will be randomized in a 1:1:1:1 ratio to receive 21 sessions of 600 pulses active iTBS, 1,200 pulses active iTBS, 1,800 pulses active iTBS, or 1,800 pulses sham iTBS in addition to conventional rehabilitation training. The primary outcome is the Fugl-Meyer Assessment of the Upper Extremity (FMA-UE) score from baseline to end of intervention, and the secondary outcomes are the Wolf Motor Function Test (WMFT), Grip Strength (GS), Modified Barthel Index (MBI), and Stroke Impact Scale (SIS). The FMA-UE, MBI, and SIS are assessed pre-treatment, post-treatment, and at the 3-weeks follow-up. The WMFT, GS, and resting-state functional magnetic resonance imaging (rs-fMRI) data will be obtained pre- and post-treatment.

**Discussion:**

The iTBS intervention in this study protocol is expected to be a potential method to promote upper limb motor recovery after stroke, and the results may provide supportive evidence for the optimal dose of iTBS intervention.

## 1. Introduction

Motor dysfunction of the upper limb occurs in 73–88% of first-ever stroke survivors, and 55–75% of patients suffer residual motor dysfunction of the upper limb 3 to 6 months after stroke onset (Lawrence et al., 2001). Stroke patients with upper limb motor dysfunction often have limited independence in daily activities and reduced quality of life (Faria-Fortini et al., 2011). Therefore, improving upper limb function is an important goal of stroke rehabilitation (Pollock et al., 2014; Veerbeek et al., 2017). To date, conventional rehabilitation training remains the primary treatment for upper limb motor dysfunction after stroke, but effectiveness is limited. It is necessary to develop other therapeutic methods to improve the effectiveness of conventional rehabilitation training for upper limb motor dysfunction after stroke (Barreca et al., 2003; Coscia et al., 2019).

In recent years, repetitive transcranial magnetic stimulation (rTMS) has been increasingly used as a safe and non-invasive neuromodulation technique for functional rehabilitation of the upper limb after stroke (Du et al., 2016; Kim et al., 2020; Ni et al., 2022; Ahmed et al., 2023). rTMS plays a therapeutic role by penetrating the skull through the magnetic field generated by the coil transient current to generate an induced current to stimulate neurons, thereby regulating cortical excitability and modulating neuroplasticity (Du et al., 2019; Brown et al., 2021; Bai et al., 2022). First applied to healthy subjects by Huang et al. (2005), theta burst stimulation (TBS) is a novel type of rTMS that can achieve similar or better results than conventional rTMS in a shorter stimulation session (Di Lazzaro et al., 2011; Blumberger et al., 2018; Si et al., 2019). Moreover, the stimulation intensity of TBS is lower, reducing the risk of adverse effects, especially seizures (Gutiérrez-Muto et al., 2020). Excitatory or inhibitory effects can be achieved by adjusting the stimulation time and interval, whereas intermittent theta burst stimulation (iTBS) has excitatory effects on the cerebral cortex (Ogata et al., 2021; Bai et al., 2023). In the iTBS, a 2 s train of TBS is repeated every 10 s for a total of 190 s (600 pulses) (Huang et al., 2005). Currently, the main target of stimulation is the primary motor cortex (M1) over the ipsilateral hemisphere when iTBS is used to treat poststroke upper limb motor dysfunction (Gao et al., 2022; Huang et al., 2022; Tang et al., 2022). Although iTBS appears to be a promising treatment for upper limb motor recovery in stroke patients, it is noteworthy that the evidence for beneficial effects of iTBS on post-stroke upper limb motor dysfunction is inconclusive. Some clinical trials have been conducted to explore the effects of iTBS on upper limb motor recovery after stroke, but the results were controversial (Talelli et al., 2012; Khan et al., 2019; Liu et al., 2019). Thus, improving the effectiveness of iTBS has become the focus of research.

One of the most important factors influencing the effectiveness of iTBS is the stimulus dose (Volz et al., 2013; Chung et al., 2016). When studying stroke patients, most researcher used a block of 600 iTBS pulses (Ackerley et al., 2014, 2016; Watanabe et al., 2018; Chen et al., 2019; Ding et al., 2021) and a few used two blocks of 600 pulses iTBS (Hsu et al., 2013; Chen et al., 2021). Previous studies in healthy human subjects suggested that iTBS might dose-dependently modulate neural plasticity, i.e., the higher the dose of iTBS, the greater the changes in neural plasticity induced (Nettekoven et al., 2014; Yu et al., 2020). Nettekoven et al. (2014) applied three blocks of 600 pulses iTBS to motor cortex and found that the cortical excitability enhancement effect was significantly greater than two blocks of 600 pulses iTBS and one block of 600 pulses iTBS. They also found that functional connectivity between M1 and ipsilateral dorsal premotor cortex continued to increase in a dose-dependent manner after 1,800 pulses of iTBS over M1. However, studies in healthy human subjects are insufficient to support the clinical application of iTBS. Other studies revealed that in patients with neurological disorders, high doses of rTMS produced stronger and longer lasting therapeutic responses than low doses (Nyffeler et al., 2009; Williams et al., 2018), but an agreement on how to select the best dose of iTBS for stroke patients with upper limb motor dysfunction is limited until now. There is a lack of studies comparing the difference in efficacy of different doses of iTBS for upper limb motor dysfunction in stroke patients.

Therefore, the objectives of this randomized controlled clinical trial are: (1) to investigate the safety and efficacy of one block of iTBS (600 pulses-iTBS), two blocks of iTBS (1,200 pulses-iTBS), or three blocks of iTBS (1,800 pulses-iTBS) in the treatment of upper limb motor dysfunction in stroke patients; (2) to compare the efficacy of three doses of iTBS; (3) to explore the neural mechanism of upper limb motor recovery after stroke using resting-state functional magnetic resonance imaging (rs-fMRI). Our hypothesis is that the higher the dose of iTBS, the greater the improvement in upper limb motor function in stroke patients. This study may provide new insights into the efficacy and underlying neuroplastic mechanisms of iTBS for treating poststroke upper limb motor dysfunction.

## 2. Methods

### 2.1. Study design

**Figure 1:**
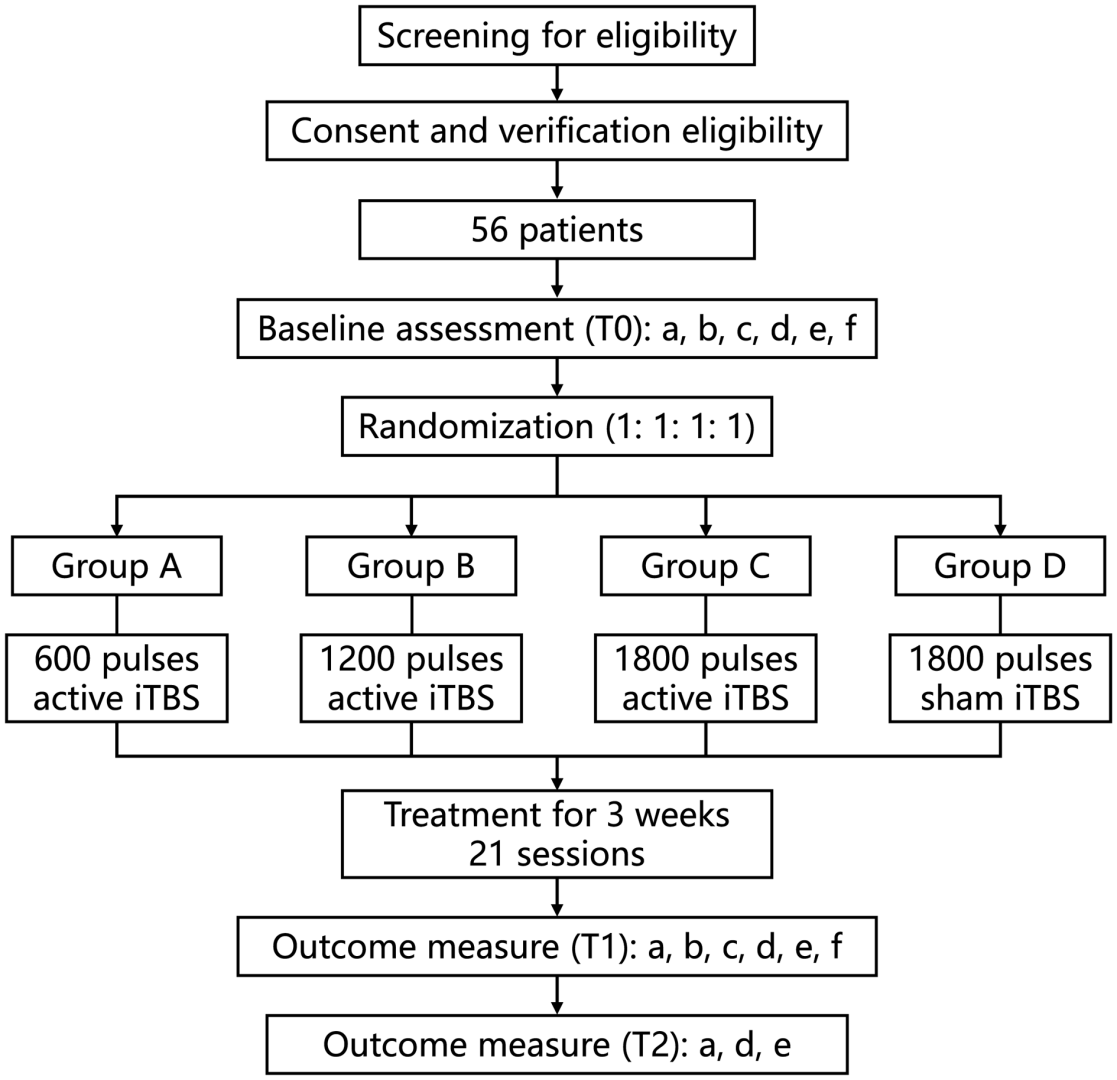
Study flow diagram. a, FMA-UE; b: WMFT; c, GS; d, MBI; e, SIS; f, MRI. iTBS, intermittent theta burst stimulation. T0, pre-treatment; T1, post-treatment; T2, at the 3-weeks follow-up.

This is a prospective, single-center, randomized, parallel, sham-controlled clinical trial. This study will be conducted at Beijing Bo’ai Hospital, China Rehabilitation Research Center (Beijing, China). Hospitalized patients will be screened in accordance with strict inclusion and exclusion criteria. After written informed consent is obtained, demographic information collection and baseline assessments are scheduled for each patient. All participants will be randomly assigned with a ratio of 1:1:1:1 to one of the following four groups: Group A, Group B, Group C, and Group D. Details of the study design and data collection are presented in Figure 1 and Table 1. Behavioral assessments will be performed at baseline (T0), at the end of the iTBS intervention (T1), and 3 weeks post-treatment (T2) to assess upper limb motor function, activities of daily living (ADLs) performance, and quality of life. In addition, rs-fMRI data will be collected before and after the end of the intervention. This study has been approved by the Medical Ethics Committee of China Rehabilitation Research Center (No.: 2022-149-01) and registered on www.chictr.org.cn under the registration number ChiCTR2300068177.

**Table 1:** Overview of data collection and study timings.

Study period	Pre-enrollment (−7 d)	T0 (−3 to 0 d)	Intervention (0–21 d)	T1 (21 + 3 d)	T2 (42 ± 3 d)
Screening	×				
Enrollment	×				
Informed consent	×				
Random allocation		×			
Demographic information		×			
FMA-UE		×		×	×
WMFT		×		×	
GS		×		×	
MAS	×	×		×	
MBI		×		×	×
SIS		×		×	×
MRI		×		×	×
Adverse event		×	×	×	×

### 2.2. Participants

#### 2.2.1. Inclusion criteria

Participants are considered eligible if they meet the following criteria: (1) have a unilateral ischemic or hemorrhagic subcortical stroke; (2) first or previous stroke without residual disability (modified Rankin Scale ≤1 before this onset); (3) 1–6 months post-stroke; (4) are 35–75 years old; (5) presented with a unilateral upper limb motor dysfunction with a Brunnstrom stage score between 2 and 5 in the upper limb or hand; (6) can sign an informed consent form.

#### 2.2.2. Exclusion criteria

Participants will be excluded from the study if they meet the following criteria: (1) have a neurological impairment, upper limb limitation, amputation, joint swelling, contracture, severe pain, or other hand condition caused by a subtentorial stroke, brain tumor, brain trauma, fracture, or other disease; (2) the modified Ashworth scores (MAS) for the shoulder, elbow, wrist, and fingers of the affected upper extremity are all in the 3–4 range; (3) contraindications involving rTMS and rs-fMRI (e.g., skin damage at the stimulation site, claustrophobia, epilepsy history, intracranial implant, cardiac pacemaker); (4) have a disturbance of consciousness (National Institute of Health stroke scale (NIHSS) 1(a) ≥1); (5) have a malignant hypertension; (6) have a malignant tumor or any cause other than a stroke that results in a life expectancy of less than 1 year; (7) have severe aphasia (NIHSS language item ≥2), dysarthria (NIHSS dysarthria item ≥2), cognitive impairment (Mini-mental State Examination (MMSE) score ≤20), deafness, etc., such that they are unable to communicate, understand or follow instructions, and cannot cooperate with treatment and assessment; (8) have a major depressive disorder or anxiety disorder (Hamilton Depression Scale (HAMD)-17 score ≥18; Hamilton Anxiety Scale (HAMA)-score ≥21) or have been diagnosed with another mental disorder; (9) medication of antidepressants or benzodiazepines; (10) have severe sensory impairment (NIHSS sensory items = 2) or severe neglect (NIHSS neglect items = 2); (11) received neuromodulation therapy such as rTMS, transcranial electrical stimulation, and transcranial focused ultrasound within 3 months prior to enrollment; (12) have a history of alcohol or drug abuse; (13) are ineligible for participation in this trial due to other examination abnormalities; (14) are pregnant, or plan to get pregnant; (15) are participating in other clinical trials.

#### 2.2.3. Withdrawal criteria

Participants will be asked to withdraw from this trial if they meet the following criteria: (1) other neuromodulation therapies similar to iTBS are added during the study period; (2) miss iTBS treatment for 3 or more consecutive days or not complete treatment within 26 days; (3) have a sudden worsening of a stroke or other serious medical condition during the study period; (4) refuse to continue treatment; (5) the iTBS protocol is arbitrarily changed; (6) lost visits.

### 2.3. Sample size

According to a previously published meta-analysis comparing iTBS and sham stimulation (Zhang et al., 2017), the expected effect size Cohen’s d for the comparison of the two groups was 0.6, and the effect size (f) for the comparison of the corresponding four groups was approximately 0.3. Using G*Power v3.1.9.2, the estimated sample size required for a 4-group design with a ratio of 1:1:1:1, given a power of 0.95 and a 2-tailed alpha error probability of 0.05, is 44 patients in total. Considering a dropout rate of 20%, at least 14 participants are needed for each group. Therefore, 56 patients will be enrolled in this study.

### 2.4. Randomization and blinding

Block randomization method will be used in this study. Once informed consent and baseline data have been obtained, all eligible participants will be randomly assigned to four groups using a random number sequence generated by SPSS 25.0 software. Random assignment is carried out by an independent researcher and kept in a sealed opaque envelope. The researchers responsible for iTBS will not be involved in the study design, recruitment, randomization, assessment, or data analysis. Participants and other researchers (such as outcome assessors or statisticians) will be blinded to the group allocation.

### 2.5. Interventions

**Figure 2:**
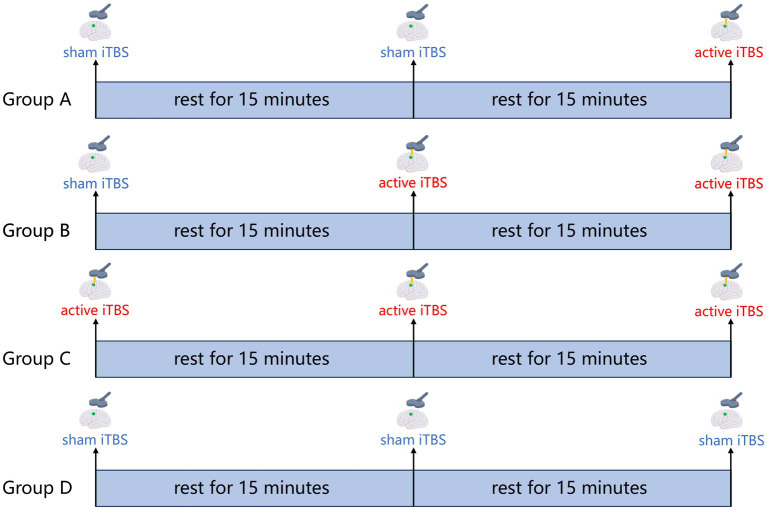
iTBS treatment protocol. Group A: 600 pulses sham iTBS + 15 min rest + 600 pulses sham iTBS + 15 min rest + 600 pulses active iTBS; Group B: 600 pulses sham iTBS + 15 min rest + 600 pulses active iTBS + 15 min rest + 600 pulses active iTBS; Group C: 600 pulses active iTBS + 15 min rest + 600 pulses active iTBS + 15 min rest + 600 pulses active iTBS; Group D: 600 pulses sham iTBS + 15 min rest + 600 pulses sham iTBS + 15 min rest + 600 pulses sham iTBS.

A transcranial magnetic stimulator (Neurosoft LLC, 5, Voronin str., Ivanovo, 153032, Russia) equipped with a figure-of-eight coil (7 cm in diameter) is used to perform the iTBS procedure. An active stimulation coil is used to deliver the active stimulation, and a sham stimulation coil is used to deliver the sham stimulation. Prior to intervention, resting motor threshold (RMT) is determined by applying a single pulse of TMS over the contralateral M1 (Tscherpel et al., 2020). RMT is defined as the minimum stimulus intensity to elicit a motor evoked potential (MEP) of 50 μV in at least 5 out of 10 consecutive trials in the relaxed abductor pollicis brevis muscle. In addition, the participants will undergo magnetic resonance scans. Similar to a previous study from our group, in this study, we will first iteratively segment the cerebral cortex of each patient into 18 functional networks based on the rs-fMRI data for each patient, thereby defining the Upper Extremity Sensorimotor Network (UESN) and the confidence values for each vertex in the UESN. Considering that TMS is likely to have the strongest influence on the gyrus crown, we exclude vertices located in the sulci, and then determine the individualized stimulation target within the UESN of the ipsilateral hemisphere on the basis of the highest confidence value. Then, according to the results of the randomized assignment, each patient will receive the navigated iTBS treatment once a day for 21 consecutive days for his or her group. The iTBS paradigm of three 50 Hz pulses repeated at 5 Hz frequency will be used, with each stimulus 2 s followed by 8 s rest, for a total of 600 pulses over 200 s. The stimulation target will be verified and maintained by a frameless neuro-navigation system (Brainsight 2; Rogue Research, Montreal, QC, Canada). The stimulation intensity is determined to be 90%RMT. The iTBS treatment in the four groups is shown in Figure 2.

Following daily iTBS intervention, each participant will undergo conventional occupational therapy and physical therapy for 1 h. The daily rehabilitation programs consist of exercises designed to promote voluntary motor recovery, including muscle stretching, passive and passive-assisted mobilization, progressive neuromuscular facilitation training, and task-oriented training. All exercises are performed by the same experienced therapists, who are unaware of the group allocation.

### 2.6. Outcome measures

The Fugl-Meyer Assessment of the Upper Extremity (FMA-UE) score from baseline (T0) to end of intervention (T1) is defined as the primary outcome. The FMA-UE is a standardized scale of upper limb motor impairment ranging from 0 to a maximum of 66 points (Fugl-Meyer et al., 1975; Chen Y. F. et al., 2022). Secondary outcomes are the Wolf Motor Function Test (WMFT), Grip Strength (GS), MAS, Modified Barthel Index (MBI), and Stroke Impact Scale (SIS). The WMFT consists of 15 tasks, each of which must be completed in at least 120 s, and the performance on each task is scored on a six-point scale (0–5) (Morris et al., 2001; Wolf et al., 2001). The GS is measured with a grip dynamometer. During the measurement, the patient’s shoulder joint is abducted to 90°, the elbow joint is flexed to 90°, and the forearm is placed on the table. The patient is instructed to hold the grip dynamometer as hard as possible (Bertrand et al., 2015). The grip dynamometer automatically records the highest value. A total of 3 measurements are taken with a 1-min rest interval between each measurement. The MAS is commonly used to assess spasticity. The MAS scores for the shoulder, elbow, wrist, and fingers of the affected upper extremity will be recorded. The MBI is used to assess the patient’s ability to perform activities of daily living (ADL), including 10 items out of 100 points (Ohura et al., 2017). The SIS is one of the commonly used scales to evaluate the quality of life of stroke patients, which consists of eight items, and each item has a full score of 100 points (Mulder and Nijland, 2016), and we will take the average score of the eight items as the outcome. Each participant will undergo the following clinical assessments pre-treatment (T0), post-treatment (T1), and at the 3-weeks follow-up (T2): FMA-UE, MBI, SIS. The following clinical assessments will be performed only before and after treatment: WMFT, GS, MAS. We will also perform exploratory analyses based on the rs-fMRI data collected before and after the treatment.

The magnetic resonance imaging (MRI) data will be collected by using a Philips Ingenia 3.0 T Magnetic Resonance System at the China Rehabilitation Research Center. Three rs-fMRI sessions of 8 min 6 s each are acquired using a gradient echo planar imaging sequence with the following parameters: TR = 2,000 ms, TE = 30 ms, FOV = 224 × 224 mm^2^, Flip angle = 90°, matrix = 64 × 64, Slice number = 32, voxel size = 3.5 × 3.5 × 4.35 mm^3^. Anatomical images are acquired using a high-resolution T1-weighted sequence (TR = 7.13 ms, TE = 3.22 ms, Flip angle = 7°, 192 slices, FOV = 256 × 256 mm^2^, Matrix = 256 × 256, voxel size = 1 × 1 × 1 mm^3^).

### 2.7. Data analysis

We will use SPSS version 25 for statistical analysis. Analysis of variance (continuous and ordinal data) or χ^2^ tests (categorical data) will be used to compare demographic and baseline characteristics. A mixed-design ANOVA (between-subjects factor: group; within-subjects factor: time; and time-by-group interaction) will be used to detect any significant differences in the change of outcomes. Any factor with a significant difference between the groups at the baseline will be included as a covariate in the ANOVAs. If a significant time-by-group interaction effect is found, comparisons using pairwise *t*-tests will be performed by separately comparing the changes from baseline. Bonferroni correction is applied for *post hoc* tests. Before entering the data into the ANOVAs, the Shapiro–Wilk test will be used to test whether the data are normally distributed. For non-normally distributed measures, the non-parametric Kruskal-Wallis H test is performed to compare across conditions. The level of significance is defined as *p* < 0.05 (2-tailed). For missing data, intention-to-treat (ITT) analysis is used (Matilde Sanchez and Chen, 2006).

The pre-processing of the MRI data will be in accordance with the previously described pipeline (Yeo et al., 2011). After preprocessing, regional homogeneity (ReHo), degree centrality (DC), amplitude of low frequency fluctuations (ALFF), and seed-to-voxel analyses will be performed to measure functional connectivity within motor networks. ReHo is calculated based on the Kendall’s concordance coefficient, which is used to measure the time-series synchronization between a given voxel and neighboring voxels, with higher values representing better concordance between the local voxel and the regional brain activities. In network analysis, DC is the most direct measure of node centrality. A higher degree of a node means that the node has a higher DC and the node is more important in the network. The ALFF value is related to the strength of local neural activity. It is often used to measure the BOLD signal in the frequency range of 0.01–0.1 Hz (Zang et al., 2004). Differences between conditions are considered significant if *p* < 0.05 at the voxel level. A false discovery rate (FDR) cluster corrected *p* < 0.05 is applied.

### 2.8. Safety

Any adverse effects occurring during MRI scan will be reported within 24 h. Participants will be provided with earplugs to protect their hearing from noise. iTBS treatment may cause headaches, scalp sensations or nociception, dizziness, or fatigue in some patients, which may resolve on their own after stimulation stops without special treatment. In particular, seizures, which are the most serious TMS-related adverse effect with a risk of about 0.02%, are expected to occur only during or immediately after stimulation (Rossi et al., 2009). To minimize the risk of adverse events, participants will be screened strictly according to the inclusion and exclusion criteria. There will be a record of all adverse events and a comparison of the incidence of adverse events between groups. In addition, serious adverse events will be reported to the ethics committee immediately upon occurrence.

## 3. Discussion

This randomized controlled clinical trial aims to investigate and compare the efficacy of three doses of iTBS on the recovery of upper limb motor function in stroke patients. Furthermore, we hope to provide a theoretical basis for clinical application by investigating the neural effects of iTBS intervention on upper limb motor dysfunction. Our results may provide supportive evidence for the optimal dose of excitatory iTBS intervention in the treatment of upper limb motor dysfunction after stroke.

In this study, we hypothesize that iTBS can promote upper limb motor recovery in stroke patients by modulating functional connectivity within the motor network. We also hypothesize that the higher the stimulus dose, the greater the improvement in motor function. The following evidence supports this hypothesis. Increasingly, it is thought that increasing the dose of rehabilitation may lead to better motor function outcomes in stroke patients (Cooke et al., 2010; Lang et al., 2015; Winstein et al., 2019). rTMS is an important physiotherapeutic approach, and its use in rehabilitation is inseparable from the choice of parameters, with the stimulus dose being of particular importance. and rTMS has gained attention in recent years as a promising physical therapy approach. iTBS is a type of rTMS that has great potential in the treatment of post-stroke motor dysfunction and has attracted much attention in recent years. Previous studies on healthy subjects have found that iTBS with more stimulus pulses more significantly enhances motor cortex excitability and functional connectivity within the motor network. However, studies comparing the effects of different doses of iTBS on upper limb motor recovery in stroke patients are lacking, and our study will fill this gap.

In terms of experiment design, our study raises some methodological concerns worth discussing. We set up four groups (Group A, Group B, Group C, and Group D) corresponding to four conditions (600 pulses active iTBS, 1,200 pulses active iTBS, 1,800 pulses active iTBS and 1,800 pulses sham iTBS) in this study. It is important to note that, for statistical reasons, the iTBS intervention protocol for both Groups A and B includes sham stimulation so that the total stimulation time per day is the same for each group, thus ensuring successful blinding. Referring to a previous similar study (Blumberger et al., 2021), and considering that patients will receive conventional rehabilitation training after iTBS intervention, we place the sham stimulation before the active stimulation in Groups A and B in order to ensure that the time interval between the last active stimulation and the conventional rehabilitation training is the same for each patient per day. In this study, block randomization is used to balance the effect of enrollment time on patients’ baseline characteristics (Lim and In, 2019). Also, outcome will be evaluated at pre-intervention, the end of first, second, and third week of post-intervention. Taking into account the length of the patient’s stay, we are going to conduct a three-week intervention with the patient. Additionally, to observe the short-term and long-term effects of iTBS, clinical outcomes will be evaluated before, after, and during the third week after the intervention.

M1 plays an important role in motor recovery after stroke. The researchers examined the brain activation during movement of the upper limb in stroke patients and described the altered activity in the ipsilateral and contralateral M1 (Rehme et al., 2012; Favre et al., 2014; Hannanu et al., 2017; Braaß et al., 2023). Previous studies have suggested that the activity of the ipsilateral M1 may be reduced and the activity of the contralateral M1 may be increased after stroke (Calautti et al., 2007, 2010; Larivière et al., 2018). Thus, non-invasive brain stimulation may be applied to modulate stroke-induced changes of motor network activity and connectivity to improve motor function (Grefkes and Fink, 2012; Tang et al., 2015). On the basis of the interhemispheric inhibition (IHI) model (Di Pino et al., 2014), excitatory stimulation applied to the ipsilateral hemisphere or inhibitory stimulation applied to the contralateral hemisphere may produce a consistent effect on functional recovery. In the present study protocol, based on baseline rs-fMRI data, we will delineate 18 functional networks for each patient, and the excitatory iTBS intervention will target M1 in the ipsilateral upper extremity sensorimotor network.

fMRI, a non-invasive brain imaging technique with high spatial resolution, has been widely used to explore the brain. Some previous studies have used fMRI to investigate the neural mechanisms by which low-frequency rTMS or high-frequency rTMS improves motor recovery after stroke (Grefkes et al., 2010; Li et al., 2016; Du et al., 2019; Guo et al., 2021; Chen Q. et al., 2022; Juan et al., 2022), but few studies have used fMRI to investigate the potential effects of iTBS on motor function after stroke. Volz et al. (2016) found that the addition of iTBS to prime physiotherapy in recovering stroke patients seemed to interfere with motor network degradation. Therefore, our study will use rs-fMRI to explore potential therapeutic mechanisms for iTBS. Although task-state fMRI can reflect brain activity during the performance of specific tasks, given that the subjects are patients with post-stroke motor dysfunction, the quality of the tasks performed by the patients may vary widely, which could affect the results, so we chose rs-fMRI.

iTBS has the advantages of easy operation and short stimulation time, and its efficacy may be influenced by the stimulus dose. We hope that our protocol and results can elucidate this potential effect and provide guidance for the treatment of patients with upper limb motor dysfunction after stroke.

## Ethics statement

This study involving human subjects was reviewed and approved by the Medical Ethics Committee of China Rehabilitation Research Center. Patients/participants will provide written informed consent to participate in this study.

## Author contributions

ZT: Supervision, Writing – original draft, Writing – review & editing. TL: Investigation, Resources, Writing – review & editing. YL: Investigation, Resources, Writing – review & editing. KH: Methodology, Writing – review & editing. WS: Visualization, Resources, Writing – review & editing. JZ: Resources, Writing – review & editing. QC: Funding acquisition, Investigation, Writing – review & editing. XZ: Writing – review & editing. HZ: Conceptualization, Writing – review & editing.
